# Changing Presenting Complaints During 72-Hour Emergency Department Revisits: Associations with Functional Status and Revisit Outcomes

**DOI:** 10.3390/healthcare14152309

**Published:** 2026-07-31

**Authors:** Wen-Chun Tsai, Hsiu-Lan Li, Yi-Shan Wu, Yig-Ting Hwang, Ming-Nan Huang, Shih-Wei Lin

**Affiliations:** 1 Department of Nursing, National Yang Ming Chiao Tung University, Taipei City 112, Taiwan; 10682@km.eck.org.tw; 2 Department of Nursing, En Chu Kong Hospital, New Taipei City 237, Taiwan; p3703hsiu@gmail.com (H.-L.L.); 13579@m.eck.org.tw (Y.-S.W.); 3 Department of Nursing, Hsin Sheng College of Medical Care and Management, Taoyuan City 325, Taiwan; 4 Department of Statistics, National Taipei University, New Taipei City 237, Taiwan; hwangyt@gm.ntpu.edu.tw; 5 Department of Emergency Medicine, En Chu Kong Hospital, New Taipei City 237, Taiwan; 01388@km.eck.org.tw; 6 Department of Information Management, Chang Gung University, Taoyuan City 333, Taiwan; 7 Department of Emergency Medicine, Keelung Chang Gung Memorial Hospital, Keelung 204, Taiwan; 8 Department of Industrial Engineering and Management, Ming Chi University of Technology, New Taipei City 243, Taiwan

**Keywords:** emergency department revisit, presenting complaint, functional status, activities of daily living, hospitalization, patient outcome

## Abstract

**Background:** Emergency department (ED) revisits within 72 h are commonly used as indicators of healthcare quality and have been associated with adverse clinical outcomes. Some patients return with presenting complaints that differ from those reported during the initial ED encounter; however, factors associated with this phenomenon have not been well described. **Methods:** This retrospective cohort study analyzed electronic health record data from a regional teaching hospital in Taiwan between 2016 and 2023. Adult patients with unscheduled ED revisits within 72 h were included. Documented presenting complaints recorded at the index visit and revisit were compared according to predefined operational definitions and categorized as consistent or changing. Multivariable logistic regression was used to identify factors associated with complaint consistency. Revisit disposition was also examined. **Results:** A total of 1957 patients met the study criteria, of whom 809 (41.3%) had changing presenting complaints. Compared with patients with consistent presenting complaints, those with changing presenting complaints were older and had lower activities of daily living (ADL) scores. In multivariable analysis, male sex was independently associated with complaint consistency (OR 1.41, *p* = 0.002), whereas increasing age was associated with a lower likelihood of consistency (OR 0.99 per year, *p* < 0.001). Higher ADL scores were also associated with complaint consistency (OR 1.04 per point, *p* = 0.023). Revisit disposition distributions differed significantly between the two groups (*p* = 0.005). In the unadjusted analysis, patients with changing documented presenting complaints were more frequently hospitalized, transferred, or died during the revisit than patients with consistent documented presenting complaints. **Conclusions:** Changing documented presenting complaints were associated with older age and poorer functional status. An unadjusted difference in revisit disposition distributions was also observed between the two groups. These findings are hypothesis-generating and require prospective validation before their clinical utility can be determined.

## 1. Introduction

Emergency department (ED) revisits within 72 h are widely used as indicators of healthcare quality and are associated with increased hospitalization, diagnostic-related adverse events, and healthcare utilization [1,2,3,4]. Although some revisits reflect progression of the original illness, others may be related to unresolved symptoms, evolving clinical conditions, or newly emerging health problems. Identifying characteristics associated with early ED revisits remains important because these encounters frequently require reassessment and may involve different clinical trajectories from the initial visit.

Previous investigations of 72 h ED revisits have focused primarily on revisit frequency, risk factors for return visits, hospital admission, and other adverse outcomes [5,6,7,8]. In contrast, little attention has been given to whether patients present with the same complaints at both encounters. Presenting complaints are fundamental to triage prioritization and diagnostic evaluation, yet the stability of complaint patterns between the index visit and an early revisit has rarely been examined.

When patients return with complaints that differ from those documented during the initial encounter, clinical reassessment may become more challenging. A change in documented presenting complaints may reflect progression of the original disease process, the development of additional symptoms, or the emergence of a new clinical condition [6,9,10]. Variations in symptom perception, communication, cognitive status, and clinical documentation may also influence how presenting complaints are recorded across encounters.

Patient-related factors may contribute to variation in complaint patterns. Older adults frequently present with atypical or nonspecific symptoms, making symptom recognition and clinical assessment more complex [11,12,13]. Functional status may also be relevant. Individuals with impaired activities of daily living (ADL) often experience frailty, multimorbidity, and greater dependence on caregivers, factors that can affect symptom recognition, communication, and healthcare-seeking behavior during acute illness [14,15,16]. These characteristics may influence how symptoms are recognized and communicated and, consequently, how presenting complaints are documented across repeated healthcare encounters.

Despite the potential relevance of changes in documented presenting complaints during early ED revisits, evidence regarding factors associated with these changes remains limited. Furthermore, it is unclear whether patients with changing presenting complaints have different revisit disposition distributions from those with consistent presenting complaints [1,17,18]. Examining these relationships may improve understanding of how documented complaint patterns differ between repeated ED encounters.

Therefore, this study aimed to identify factors associated with changes in documented presenting complaints between an index ED visit and a 72 h revisit and to examine the unadjusted relationship between presenting complaint patterns and revisit disposition, including hospitalization, transfer, and death.

## 2. Methods

### 2.1. Study Design and Setting

This retrospective cohort study was conducted in the emergency department (ED) of a regional teaching hospital in Taiwan, which manages approximately 60,000 ED visits annually. The ED operates a nurse-led triage system supported by structured electronic health records (EHRs). Patients who returned to the ED within 72 h of a previous visit were identified through the EHR database, and consecutive encounters occurring within this interval were linked at the patient level.

The study was approved by the Institutional Review Board of En Chu Kong Hospital (ECKIRB1130405) and was conducted in accordance with the Declaration of Helsinki. Because the study used de-identified retrospective data, the requirement for informed consent was waived.

### 2.2. Data Source and Patient Selection

All ED encounters recorded between 1 January 2016 and 31 December 2023 were screened. Eligible patients were adults who returned to the ED within 72 h after discharge from an index ED visit. Both direct presentations and referrals from outside institutions were included.

The EHR database contained routinely collected demographic information, lifestyle characteristics, anthropometric measurements, functional status, level of consciousness, medical history, and initial triage acuity. Initial triage acuity was assessed using the Taiwan Triage and Acuity Scale (TTAS), a standardized five-level triage system routinely used in Taiwanese emergency departments. During the study period, 1960 patients met the revisit criteria. Three patients were excluded because of missing key assessment variables, including demographic data, lifestyle information, anthropometric measures, or activities of daily living (ADL) scores. The final analytic cohort consisted of 1957 patients.

**Figure 1:**
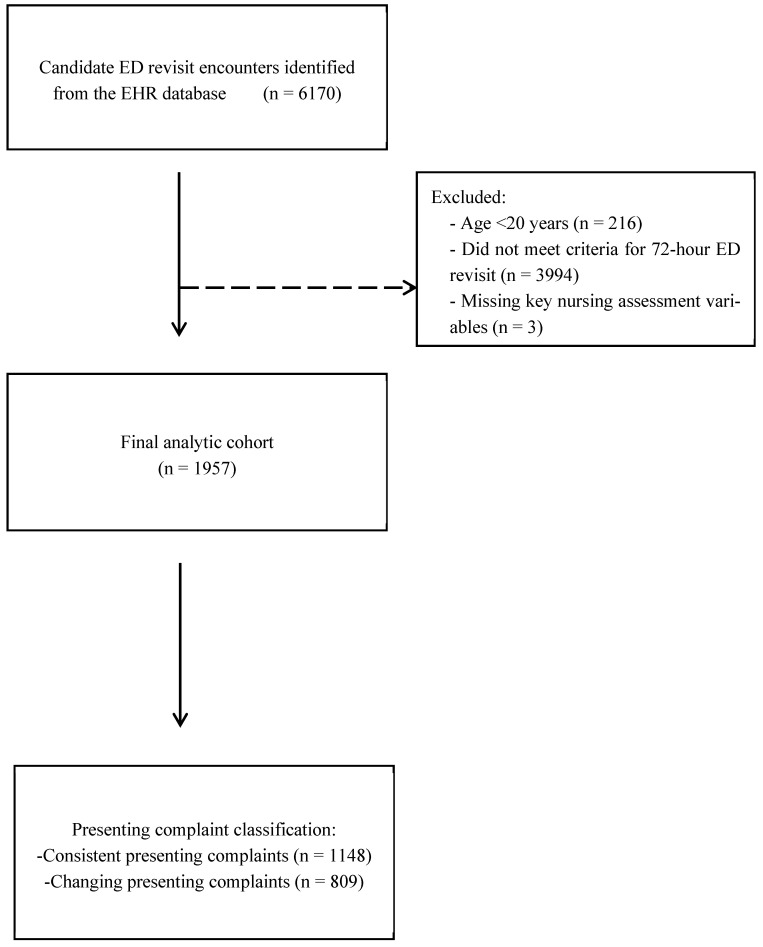
Flow diagram of patient selection. Patients with unscheduled emergency department revisits within 72 h were identified from the electronic health record database. Cases with missing key nursing assessment variables were excluded prior to analysis. Patients were subsequently categorized into consistent and changing presenting complaint groups by comparing the documented presenting complaints at the index visit and the revisit according to the prespecified operational definitions.

Additional variables included Glasgow Coma Scale (GCS) score, medical history, surgical history, and routine ED assessment findings. Missing data accounted for less than 5% of observations; therefore, complete-case analysis was performed without imputation. The patient selection process is shown in Figure 1.

### 2.3. Outcome Measures

The primary outcome was the consistency of documented presenting complaints between the index ED visit and the 72 h revisit. At each ED visit, the presenting complaint was initially documented by the triage nurse based on the patient’s reported reason for seeking care and was subsequently reviewed and confirmed by the treating emergency physician during clinical assessment. The final presenting complaint recorded in the electronic health record was used for analysis. The documented presenting complaints at the two visits were compared according to prespecified operational definitions and categorized as consistent when they represented the same or clinically related presenting complaint categories and as changing when they represented clinically unrelated categories. No additional retrospective adjudication or reclassification was performed by the investigators.

The secondary outcome was disposition after the revisit, categorized as discharge, hospitalization, transfer, or death.

### 2.4. Statistical Analysis

Statistical analyses were performed using SAS version 9.4 (SAS Institute, Cary, NC, USA). Continuous variables were presented as mean ± standard deviation (SD), and categorical variables as frequencies and percentages. Baseline characteristics were compared between patients with consistent and changing presenting complaints. Continuous variables were analyzed using independent-sample *t*-tests, whereas categorical variables were compared using chi-square tests or Fisher’s exact tests when appropriate. Demographic, socioeconomic, lifestyle, and functional status variables were examined in the multivariable analysis. Initial triage acuity was evaluated as a candidate covariate because it may reflect illness severity at the index visit. However, it was not retained in the final multivariable model because it was not independently associated with the outcome and did not materially affect the estimates of the primary variables. Variables with sparse distributions or limited clinical interpretability were excluded from the final model. Multicollinearity was assessed before model fitting and was not considered problematic. Adjusted odds ratios (ORs) with 95% confidence intervals (CIs) were calculated. Model discrimination was assessed using the C-statistic. Statistical significance was defined as a two-sided *p*-value < 0.05.

## 3. Results

### 3.1. Baseline Characteristics

Among 1957 patients with 72 h ED revisits, 809 (41.3%) had changing presenting complaints and 1148 (58.7%) had consistent presenting complaints between encounters. Patients with changing presenting complaints were, on average, older than those with consistent complaints (65.25 ± 17.94 vs. 59.34 ± 18.24 years, *p* < 0.001). ADL scores were lower among patients with changing presenting complaints (17.08 ± 3.32 vs. 17.86 ± 2.68, *p* < 0.001). They were also shorter and weighed less than patients with consistent complaints (both *p* ≤ 0.001). No significant difference was observed in BMI (*p* = 0.289).

Continuous baseline characteristics are shown in Table 1.

### 3.2. Categorical Variables

Group differences were also seen in several categorical variables. Women had a higher proportion of changing presenting complaints than men (46.74% vs. 37.25%, *p* < 0.001). Revisit interval differed between groups (*p* = 0.014), with changing presenting complaints occurring more often among patients returning after longer intervals. Educational level was associated with complaint pattern (*p* < 0.001). Changing presenting complaints were more common among patients with lower educational attainment. Smoking (*p* = 0.001), alcohol use (*p* = 0.019), and betel nut use (*p* = 0.047) also differed between groups. Across all ADL domains, patients with partial or complete dependence were more likely to present with changing presenting complaints than those who were independent. Significant differences were observed for bathing, bed mobility, eating, toileting, and cane use (all *p* < 0.001). No group differences were found for BMI category, living arrangement, marital status, sleep duration, diet type, or previous surgery (all *p* > 0.05).

Categorical characteristics are summarized in Table 2.

### 3.3. Presenting Complaint Category Transitions

Presenting complaint patterns were not uniformly maintained between visits. Examination of presenting complaint category transitions showed that respiratory, infectious, and nonspecific complaint categories were more frequently followed by a different category at the revisit. In contrast, circulatory disease and genitourinary disease demonstrated greater category consistency between encounters.

Transitions between presenting complaint categories are shown in Table 3.

### 3.4. Revisit Disposition

The proportion of patients with changing presenting complaints increased across increasingly severe revisit dispositions. Changing presenting complaints were observed in 39.34% of patients discharged from the ED, 42.05% of hospitalized patients, 49.04% of transferred patients, and 65.22% of patients who died during the revisit encounter (*p* = 0.005).

### 3.5. Multivariable Analysis

Multivariable logistic regression identified several factors independently associated with complaint consistency. Male sex was associated with greater odds of complaint consistency (OR 1.41, *p* = 0.002), whereas increasing age was associated with a lower likelihood of consistency (OR 0.99, *p* < 0.001). Higher ADL scores were also associated with complaint consistency (OR 1.04, *p* = 0.023). None of the remaining variables retained statistical significance after adjustment. Model discrimination was modest (C-statistic = 0.62).

The multivariable logistic regression results are presented in Table 4.

## 4. Discussion

Older age was independently associated with changing presenting complaints. Older adults often present with nonspecific or evolving symptoms, making initial clinical assessment more challenging. As disease progression becomes more apparent over time, documented presenting complaints may differ between the index visit and the revisit. As clinical conditions evolve, presenting complaint patterns may become more apparent or clinically distinct, contributing to differences in documented presenting complaints between visits [11,12,13]. Although the adjusted odds ratio per 1-year increase in age was close to 1.0, the cumulative association over a 10-year age difference corresponded to approximately 10% lower odds of having consistent documented presenting complaints. This finding should be interpreted cautiously because the overall effect size was modest.

Lower ADL scores were associated with changing presenting complaints. Functional impairment may influence symptom recognition, communication, and healthcare-seeking behavior. Dependence on caregivers and the presence of multiple chronic conditions may further affect how symptoms are perceived and reported during acute illness [14,15,16]. Although each one-point increase in ADL score was associated with only a modest increase in the odds of complaint consistency, the finding suggests that overall functional independence may contribute to more consistent documented presenting complaint patterns across repeated ED visits.

Patients with changing documented presenting complaints were more frequently hospitalized, transferred, or died during the revisit in the unadjusted analysis. However, revisit disposition was examined descriptively and was not modeled as a dependent outcome. This finding should therefore not be interpreted as evidence that changes in presenting complaints independently predict hospitalization, transfer, or death. Rather, the observed difference should be considered hypothesis-generating and requires prospective validation.

Previous studies of early ED revisits have primarily focused on revisit rates, hospital admission, and adverse outcomes [5,6,7,8,9,19]. Comparatively little attention has been given to whether presenting complaints remain stable between encounters. In clinical practice, reassessment often begins with the patient’s current complaint. When the presenting complaint differs from that recorded during the index visit, clinicians may need to broaden the differential diagnosis and reconsider whether the patient’s condition has evolved since the original evaluation.

The model had limited discrimination (C-statistic = 0.62), indicating that the included variables did not fully explain presenting complaint consistency. Factors such as cognitive impairment, caregiver involvement, communication barriers, and disease progression may contribute to how symptoms are perceived, described, and documented during repeat ED encounters [20,21,22]. Future prospective studies incorporating cognitive impairment, caregiver involvement, communication barriers, and disease progression are needed to determine whether changes in documented presenting complaints provide independent clinical information beyond routinely available patient characteristics.

Several limitations should be considered. First, this retrospective single-center study may have limited generalizability. Second, presenting complaints were extracted from routine electronic health records rather than independently adjudicated by the investigators. The comparison between documented presenting complaints at the index visit and the revisit was performed according to prespecified operational definitions. Because no independent retrospective adjudication was performed, inter-rater reliability could not be assessed, and some variability in routine clinical documentation and complaint categorization cannot be excluded. Third, information on cognitive impairment, health literacy, caregiver involvement, and communication barriers was unavailable. Finally, the model had limited discrimination, indicating that relevant factors associated with documented presenting complaint consistency.

## 5. Conclusions

Changing documented presenting complaints were associated with older age and poorer functional status. An unadjusted difference in revisit disposition distributions was also observed between the consistent and changing presenting complaint groups. These findings should be considered hypothesis-generating and require prospective validation before changes in documented presenting complaints can be considered clinically useful.

## Figures and Tables

**Table 1:** Continuous baseline characteristics by presenting complaint pattern.

Variable	Consistent Presenting Complaints Mean ± SD	Changing Presenting Complaints Mean ± SD	Total Mean ± SD	*p*-Value
ADL score	17.86 ± 2.68	17.08 ± 3.32	17.54 ± 2.99	<0.001
Age, years	59.34 ± 18.24	65.25 ± 17.94	61.79 ± 18.34	<0.001
BMI, kg/m^2^	24.75 ± 4.69	24.51 ± 5.02	24.65 ± 4.83	0.289
Height, cm	162.70 ± 9.20	160.50 ± 9.44	161.80 ± 9.36	<0.001
Weight, kg	65.90 ± 15.41	63.46 ± 15.41	64.90 ± 15.45	0.001

Table note: Values are presented as mean ± standard deviation. ADL, activities of daily living; BMI, body mass index. *p*-values were calculated using independent-sample *t*-tests.

**Table 2:** Categorical baseline characteristics by presenting complaint pattern.

Variable	Category	Changing Presenting Complaints n (%)	Consistent Presenting Complaints n (%)	Total n (%)	*p*-Value
Sex	Female	394 (46.74)	449 (53.26)	843 (43.08)	<0.001
	Male	415 (37.25)	699 (62.75)	1114 (56.92)	
BMI category	Underweight	122 (44.20)	154 (55.80)	276 (14.10)	0.573
	Normal	301 (41.01)	433 (58.99)	734 (37.51)	
	Overweight	191 (42.35)	260 (57.65)	451 (23.05)	
	Obese	195 (39.31)	301 (60.69)	496 (25.34)	
Revisit disposition	Hospitalized	726 (41.72)	1014 (58.28)	1740 (88.91)	0.327
	Discharged	83 (38.25)	134 (61.75)	217 (11.09)	
Revisit interval, h	≤24	368 (38.06)	599 (61.94)	967 (49.41)	0.014
	24–48	267 (44.50)	333 (55.50)	600 (30.66)	
	>48	174 (44.62)	216 (55.38)	390 (19.93)	
Education	Illiterate	94 (51.09)	90 (48.91)	184 (9.45)	<0.001
	Elementary	272 (47.89)	296 (52.11)	568 (29.16)	
	Junior high	145 (38.06)	236 (61.94)	381 (19.56)	
	High school or above	292 (35.83)	523 (64.17)	815 (41.84)	
Living arrangement	With family	729 (41.21)	1040 (58.79)	1769 (90.39)	0.808
	Living alone	46 (40.35)	68 (59.65)	114 (5.83)	
	Long-term care	24 (48.00)	26 (52.00)	50 (2.55)	
	Other	10 (41.67)	14 (58.33)	24 (1.23)	
Marital status	Married	546 (40.93)	788 (59.07)	1334 (68.17)	0.591
	Other	263 (42.22)	360 (57.78)	623 (31.83)	
Smoking	Never	518 (44.05)	658 (55.95)	1176 (60.09)	0.001
	Former	155 (33.99)	301 (66.01)	456 (23.30)	
	Current	136 (41.85)	189 (58.15)	325 (16.61)	
Alcohol use	Never	598 (43.08)	790 (56.92)	1388 (70.92)	0.019
	Former	30 (29.13)	73 (70.87)	103 (5.26)	
	Occasional	58 (41.73)	81 (58.27)	139 (7.10)	
	Heavy	123 (37.61)	204 (62.39)	327 (16.71)	
Betel nut use	Never	721 (42.02)	995 (57.98)	1716 (87.69)	0.047
	Former	24 (34.78)	45 (65.22)	69 (3.53)	
	Occasional	48 (43.64)	62 (56.36)	110 (5.62)	
	Current	16 (25.81)	46 (74.19)	62 (3.17)	
Sleep duration	>8 h	608 (42.16)	834 (57.84)	1442 (73.68)	0.215
	≤8 h	201 (39.03)	314 (60.97)	515 (26.32)	
Bathing (ADL)	Independent	568 (37.87)	932 (62.13)	1500 (76.65)	<0.001
	Partially dependent	137 (51.50)	129 (48.50)	266 (13.59)	
	Fully dependent	104 (54.45)	87 (45.55)	191 (9.76)	
Bed mobility (ADL)	Independent	605 (38.22)	978 (61.78)	1583 (80.89)	<0.001
	Partially dependent	111 (52.86)	99 (47.14)	210 (10.73)	
	Fully dependent	93 (56.71)	71 (43.29)	164 (8.38)	
Eating (ADL)	Independent	641 (39.01)	1002 (60.99)	1643 (83.96)	<0.001
	Partially dependent	84 (50.00)	84 (50.00)	168 (8.58)	
	Fully dependent	84 (57.53)	62 (42.47)	146 (7.46)	
Diet type	Vegetarian	55 (40.15)	82 (59.85)	137 (7.00)	0.769
	Nonvegetarian	754 (41.43)	1066 (58.57)	1820 (93.00)	
Toileting (ADL)	Independent	566 (37.66)	937 (62.34)	1503 (76.80)	<0.001
	Partially dependent	140 (52.83)	125 (47.17)	265 (13.54)	
	Fully dependent	103 (54.50)	86 (45.50)	189 (9.66)	
Cane use (ADL)	Independent	590 (38.02)	962 (61.98)	1552 (79.31)	<0.001
	Partially dependent	124 (52.99)	110 (47.01)	234 (11.96)	
	Fully dependent	95 (55.56)	76 (44.44)	171 (8.74)	
Medication use	No	200 (37.04)	340 (62.96)	540 (27.59)	0.017
	Yes	609 (42.98)	808 (57.02)	1417 (72.41)	
Previous surgery	No	234 (41.05)	336 (58.95)	570 (29.13)	0.869
	Yes	575 (41.46)	812 (58.54)	1387 (70.87)	

Table note: Values are presented as n (%). ADL, activities of daily living; BMI, body mass index. *p*-values were calculated using chi-square tests or Fisher’s exact tests, as appropriate. Percentages represent the proportion within each category.

**Table 3:** Comparison of presenting complaint categories between the index ED visit and the 72-hour revisit.

	72-Hour Revisit																					
	Skin		MSK		Cerebrovasc		Resp		GU		GI		Nonspecific		Circ		Injury		Infect		Head/Trunk/Ext	
Index Visit	n	%	n	%	n	%	n	%	n	%	n	%	n	%	n	%	n	%	n	%	n	%
Skin	4	44.44	0	0.00	0	0.00	0	0.00	1	11.11	0	0.00	1	11.11	0	0.00	0	0.00	3	33.33	0	0.00
MSK	0	0.00	51	68.92	2	2.70	2	2.70	0	0.00	3	4.05	9	12.16	0	0.00	0	0.00	2	2.70	5	6.76
Cerebrovasc	0	0.00	0	0.00	29	65.91	5	11.36	0	0.00	1	2.27	9	20.45	0	0.00	0	0.00	0	0.00	0	0.00
Resp	1	0.62	0	0.00	1	0.62	94	58.39	8	4.97	5	3.11	28	17.39	14	8.70	0	0.00	10	6.21	0	0.00
GU	0	0.00	1	0.35	1	0.35	8	2.80	208	72.73	23	8.04	36	12.59	2	0.70	0	0.00	7	2.45	0	0.00
GI	0	0.00	2	0.38	4	0.76	12	2.28	40	7.60	391	74.33	55	10.46	11	2.09	1	0.19	6	1.14	4	0.76
Nonspecific	3	0.57	9	1.71	34	6.45	69	13.09	62	11.76	58	11.01	211	40.04	34	6.45	3	0.57	37	7.02	7	1.33
Circ	0	0.00	0	0.00	0	0.00	7	9.72	2	2.78	0	0.00	14	19.44	48	66.67	0	0.00	1	1.39	0	0.00
Infect	1	0.67	2	1.34	3	2.01	15	10.07	8	5.37	13	8.72	13	8.72	4	2.68	0	0.00	89	59.73	1	0.67
Head/Trunk/Ext	0	0.00	37	33.94	9	8.26	5	4.59	6	5.50	3	2.75	20	18.35	1	0.92	1	0.92	4	3.67	23	21.10

**Abbreviations of presenting complaint categories:** Skin = diseases of the skin and subcutaneous tissue; MSK = diseases of the musculoskeletal system and connective tissue; Cerebrovasc = other cerebrovascular diseases; Resp = diseases of the respiratory system; GU = diseases of the genitourinary system; GI = symptoms and signs involving the digestive system and abdomen; Nonspecific = symptoms, signs, and abnormal clinical and laboratory findings, not elsewhere classified; Circ = diseases of the circulatory system; Injury = injury, poisoning, and certain other consequences of external causes; Infect = certain infectious and parasitic diseases; Head/Trunk/Ext = injuries to the head, trunk, and extremities.

**Table 4:** Multivariable logistic regression analysis of factors associated with consistent presenting complaints.

Variable	Comparison	Est	SE	*p*	OR	95% CI
Intercept		0.61	0.53	0.247		
Sex	Male vs. female	0.35	0.11	0.002	1.41	1.13–1.77
Age	Per 1-year increase	−0.010	0.003	<0.001	0.99	0.98–0.99
Revisit interval (h)	24–48 vs. ≤24	−0.24	0.11	0.028	0.79	0.64–0.97
	>48 vs. ≤24	−0.18	0.12	0.144	0.83	0.65–1.06
Sleeping pattern	Abnormal vs. normal	−0.10	0.14	0.492	0.91	0.69–1.20
BMI category	Normal vs. underweight	0.03	0.15	0.837	1.03	0.77–1.38
	Overweight vs. underweight	−0.13	0.16	0.430	0.88	0.64–1.21
	Obese vs. underweight	−0.07	0.16	0.662	0.93	0.68–1.28
Education	Elementary vs. illiterate	−0.09	0.18	0.618	0.92	0.65–1.30
	Junior high vs. illiterate	−0.01	0.21	0.980	0.99	0.67–1.49
	High school or above vs. illiterate	−0.04	0.20	0.834	0.96	0.65–1.42
Betel nut use	Quit vs. never	−0.12	0.28	0.670	0.89	0.52–1.53
	Occasional vs. never	−0.31	0.21	0.144	0.73	0.48–1.11
	Yes vs. never	0.35	0.31	0.267	1.41	0.77–2.60
Smoking	Quit vs. never	0.07	0.14	0.626	1.07	0.81–1.42
	Current vs. never	0.04	0.15	0.811	1.04	0.77–1.39
Marital status	Other vs. married	−0.11	0.11	0.279	0.89	0.73–1.10
Medication use	Yes vs. no	−0.06	0.11	0.613	0.95	0.76–1.17
Surgery	Yes vs. no	0.16	0.11	0.136	1.17	0.95–1.45
Diet type	Nonvegetarian vs. vegetarian	−0.12	0.19	0.511	0.88	0.61–1.28
ADL score		0.04	0.02	0.023	1.04	1.01–1.08

**Abbreviations:** OR, odds ratio; CI, confidence interval; SE, standard error; ADL, activities of daily living; BMI, body mass index. The dependent variable was consistent presenting complaints (yes/no). Odds ratios greater than 1 indicate higher odds of having consistent presenting complaints, whereas odds ratios less than 1 indicate lower odds. Model C-statistic = 0.62.

## Data Availability

The datasets analyzed during the current study are not publicly available because they contain institutional clinical data and are subject to privacy and regulatory restrictions. De-identified data may be available from the corresponding author upon reasonable request and with permission from En Chu Kong Hospital and the Institutional Review Board.
